# Biotransformation of the Mycotoxin Enniatin B1 by CYP P450 3A4 and Potential for Drug-Drug Interactions

**DOI:** 10.3390/metabo9080158

**Published:** 2019-07-27

**Authors:** Lada Ivanova, Ilia G. Denisov, Yelena V. Grinkova, Stephen G. Sligar, Christiane K. Fæste

**Affiliations:** 1 Chemistry and Toxinology Research Group, Norwegian Veterinary Institute, P.O. Box 750 Sentrum, 0106 Oslo, Norway; 2 Department of Biochemistry, University of Illinois, Urbana, IL 61802, USA

**Keywords:** enniatin B1, deoxynivalenol, cytochrome P450 3A4, functional nanodiscs, biotransformation, inhibition, human liver microsomes

## Abstract

Enniatins (ENNs) are fungal secondary metabolites that frequently occur in grain in temperate climates. Their toxic potency is connected to their ionophoric character and lipophilicity. The biotransformation of ENNs predominantly takes place via cytochrome P450 3A (CYP 3A)-dependent oxidation reactions. Possible interaction with ENNs is relevant since CYP3A4 is the main metabolic enzyme for numerous drugs and contaminants. In the present study, we have determined the kinetic characteristics and inhibitory potential of ENNB1 in human liver microsomes (HLM) and CYP3A4-containing nanodiscs (ND). We showed in both in vitro systems that ENNB1 is mainly metabolised by CYP3A4, producing at least eleven metabolites. Moreover, ENNB1 significantly decreased the hydroxylation rates of the typical CYP3A4-substrate midazolam (MDZ). Deoxynivalenol (DON), which is the most prevalent mycotoxin in grain and usually co-occurrs with the ENNs, was not metabolised by CYP3A4 or binding to its active site. Nevertheless, DON affected the efficiency of this biotransformation pathway both in HLM and ND. The metabolite formation rates of ENNB1 and the frequently used drugs progesterone (PGS) and atorvastatin (ARVS) lactone were noticeably reduced, which indicated a certain affinity of DON to the enzyme with subsequent conformational changes. Our results emphasise the importance of drug–drug interaction studies, also with regard to natural toxins.

## 1. Introduction

**Figure 1:**
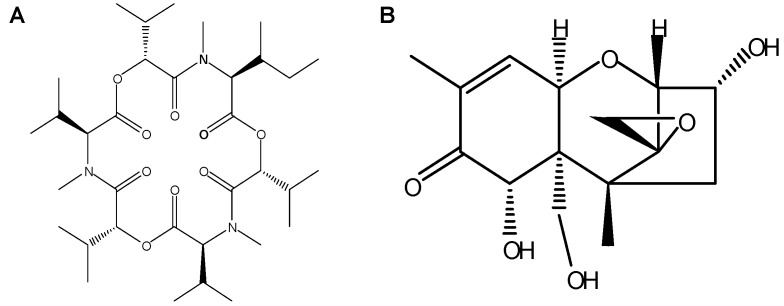
Molecular structures of (**A**). Enniatin B1 (ENNB1) and (**B**). Deoxynivalenol (DON).

Enniatins (ENNs) are fungal metabolites that occur with high frequency in grain, but for which maximum levels in food and feed have not been established by national and international food safety authorities so far [1]. In regards to their prevalence and substantial concentrations in grain-based products [2], the determination of their toxicological potency is considered to be important and necessary for the evaluation of human and animal risk from dietary exposure [3]. ENNs are six-membered cyclic depsipeptides that are mainly produced by *Fusarium* species. More than 29 ENNs analogues have been identified, among which enniatin B1 (ENNB1) is one of the most frequently detected in grains and grain-based products (Figure 1A), with a maximum recorded concentration of 5.72 mg/kg in Finnish barley [3]. Enniatin B (ENNB), enniatin A (ENNA) and enniatin A1 (ENNA1) are other important ENNs. The lipophilic ENNs have been shown to be carried-over in the food chain [2,4].

Although ENNs represent an emerging food safety issue, only limited data regarding in vivo toxicity are available. Recently, a no-observed adverse effect level (NOAEL) for ENNB has been determined in female mice (0.18 mg/kg body-weight (bw)/day) [5]. The European Food Safety Authority (EFSA) has concluded that the risk for human health from acute exposure to ENNs is low, but that there might be concern with respect to chronic exposure at lower levels [3]. Therefore, new knowledge regarding the toxic profile of the ENNs is required. On the cellular level, the cyclopeptidic ENNs form ionophores with hydrophilic groups in the core and hydrophobic groups on the outside. They can transport monovalent and divalent cations, either in sandwiched complexes or by creating channels in biological membranes, disrupting the physiological balance, which has been demonstrated in a number of in vitro test systems [4]. However, the underlying molecular mechanisms that lead to distinct cytotoxic effects are still not fully understood. ENNs are cytotoxic at low micromolar concentrations [1,4]. The observed activities include specific inhibition of acyl-coenzyme A cholesterol acyltransferase (ACAT), depolarization of mitochondria, inhibition of osteoclastic bone resorption, and induction of apoptosis in cancer cells, as well as interactions with ATP-binding cassette transporters, like *P*-glycoprotein [3]. Cells that were exposed to ENNs at toxic concentrations undergo cell cycle arrest in the G2/M phase and the loss of lysosomal and mitochondrial functionalities, which results in apoptotic and/or necrotic cell death [1]. There are conflicting data as to whether the generation of reactive oxygen species (ROS) is involved in these processes [6,7,8]. Furthermore, it is undecided whether ENNs have relevant genotoxic potential. Whereas, ENNs only caused minor DNA damage in vitro [6,9], ENNB induced a dose-dependent increase of DNA damage in liver and bone marrow after oral exposure in male mice, which gave rise to questions regarding the potential genotoxic hazard associated with this class of mycotoxins [5]. 

ENNs regularly co-occur in grain and food products with several other *Fusarium* mycotoxins, including deoxynivalenol (DON) [3] (Figure 1B), which is a potent inhibitor of protein synthesis and one of the most studied members of the large family of trichothecenes [10]. The prediction of the toxicity of mycotoxin mixtures is complicated and they cannot simply be based on a combination of the individual toxicities. Exposure to multiple contaminants may lead to additive, synergistic, or antagonistic effects [11]. Moreover, the exact character of interactions between the compounds can sometimes be difficult to define, as the experimental design of the study can have considerable influence on the result [11,12]. When the cytotoxic potentials of ENNB and DON were studied individually and as a binary combination in human colon adenocarcinoma (Caco-2) cells, the effectivities varied considerably with mycotoxin concentrations and exposure times [13]. Generally, ENNB reduced cell viability more than DON at higher concentration levels; however, concentrations below 0.625 µM produced a slight increase in cell viability when compared to non-exposed cells. The 1:1 combination of the mycotoxins resulted in a moderately antagonistic effect for concentrations below 1.25 µM and an additive to synergistic effect at concentrations above 2.5 µM after 24 and 48 h of exposure [13]. In contrast, after exposure for 72 h, the mixture was slightly synergistic at concentrations below 0.625 µM, moderately antagonistic for 1.25 µM and 2.5 µM, again synergistic at 5 µM, showing that complex, time-, and concentration-dependant molecular mechanisms are involved in generating the overall outcome. Comparable results were shown for Chinese hamster ovarian (CHO-K1) and African green monkey kidney epithelial (Vero) cells after exposure to DON and beauvericin, which is a mycotoxin with similar molecule structure to the ENNs [14]. The combined cytotoxic effectivity of mycotoxin mixtures is considered as being linked to similarities or dissimilarities in the respective modes of action, but the evident dependency on exposure times and concentration levels point at a more complex interrelation.

ENNs and DON belong to two different compound classes with specific mechanisms of action. Nevertheless, the observed combinatory effects might be attributed to interactions at common binding sites/targets in the cells. Moreover, both mycotoxins are subject to extensive hepatic metabolism. ENNs are mainly oxidised by membrane-bound cytochrome P450 3A enzymes (CYP 3A) (Phase I metabolism) [15,16], while DON is metabolised by conjugation with glucuronic acid (Phase II metabolism) that is catalysed by cytosolic glucuronyl transferases [17]. Furthermore, ENNs, as well as DON, are transported through the gastrointestinal epithelium via efflux carriers, such as *P*-glycoprotein (*P*gp), which is also known as multidrug resistance protein 1 (MDR1) [18]. Therefore it could be possible that the combinatory effects are not restricted to cytotoxic potency, but they might also moderate the biotransformation processes and oral bioavailability after in vivo uptake. The hydrophilic DON molecule can form conjugates with thiol-groups in peptides, such as glutathione [19], and also possibly with proteins, which might alter their functionality. Changes in the metabolism capacity of CYP 3A, which is one of the most important enzyme classes involved in xenobiotic metabolism, would have considerable consequences for this biotransformation pathway and the conversion rates of the respective substrates. 

Human CYP3A4 is responsible for the metabolism of numerous drugs and contaminants, and thus a major site for interactions [20]. The enzyme has multiple binding sites and inhibition, induction, and allosteric modification of reactions by interacting compounds can be observed. CYP P450 are haem-containing monooxygenases showing a characteristic spin shift of the haem iron from low-spin to high-spin state upon substrate binding, which can be used as a read-out signal in the binding studies with individual compounds and mixtures. Functional ND, self-assembled nanoscale phospholipid bilayers containing recombinant monomeric CYP3A4 and an NADPH-dependent electron donor, allow for the investigation of substrate interactions under physiological conditions [21,22]. In the ND model, the integrative membrane protein CYP3A4 is stably and homogenously solubilised, which makes quantitative analyses possible and it ensures good experimental reproducibility. The test system has been successfully used for mechanistic studies of drug-drug interactions [20,23], showing excellent applicability for a wide range of compounds. Thus, it can be safely assumed that a further broadening of the substrate range is feasible and also that interactions between mycotoxins can be studied with the ND assay. 

In the present study, we have investigated the kinetic profile of ENNB1 in human liver microsomes and interactions with other compounds that are hydroxylated by CYP3A4. Additionally, we have studied DON-induced changes in the metabolism of ENNB1 and typical CYP3A4 substrates in functional ND, with the aim of clarifying the potential metabolic interactions of these two important co-occurring mycotoxins.

## 2. Results 

### 2.1. Determination of Kinetic Parameters of ENNB1 Depletion in HLM 

ENNB1 was metabolised by HLM under conditions of first-order kinetics, with the aim of establishing kinetic in vitro data that are suitable for the prediction of in vivo parameters [24]. The depletion assay was performed with three initial concentrations of ENNB1 (0.8 µM, 3 µM, and 6 µM) for the determination of elimination constants and the extrapolation of the Michaels–Menten constant K_M_. All of the assays that were performed in the present study used concentrations below the estimated K_M_ of 10.1 µM (Table 1). ENNB1 was metabolised to a significant extent and less than 4% of the unchanged molecule was detectable after incubatiion of 6 µM for 60 min. The kinetic parameters for ENNB1 in humans after oral uptake were predicted on the basis of the half-life in assay (t_1/2, assay_) while using a substrate concentration of 3 µM. An intermediate hepatic blood clearance was estimated (CL_b_) while using the non-restrictive well-stirred model and negligising potential substance binding to microsomal proteins, as well as binding to plasma proteins (Table 1). Under the assumption of complete absorption from the gastrointestinal tract, the maximum bioavailability was predicted as 45%. However, when considering the relatively high lipophilicity of ENNB1, binding to proteins and tissue can be expected, which would reduce the in vivo CL_b_ and bioavailability (f%). 

### 2.2. Biotransformation Products of ENNB1 in HLM

After incubating ENNB1 (6 µM) for 60 min, the ENNB1-derived metabolites were analysed by LC-ITMS and characterised regarding the molecular masses and retention times, as previously described [16]. In total, 11 metabolites (M1–M11) were identified and named with respect to their appearance in the chromatogram (Supplementary Table S1; Supplementary Figure S1). The formation of the metabolites was NADPH-dependent and the metabolites were not observed in control incubations without NADPH, which indicated that ENNB1 is substrate to phase I-metabolism enzymes. The putative ENNB1 metabolites were products of hydroxylation (M2, M3/4, M5), carbonylation (M6, M7, M8), carboxylation (M9, M10, M11), and oxidative methylation (M1) reactions [16]. After 60 min incubation, the signal intensities of the different metabolites were in the order of M6 > M3/4 > M5 > M9 > M11 > M10 > M2 > M8 > M7. When considering the structural similarities between ENNB1 and its metabolites, we assumed approximately similar responses in LC-ITMS analysis, so that the relative abundances (peak areas) of the metabolites were used as indicators for the ratio of their formation. Accordingly, M3/4, M5 and M6 appeared to be the main biotransformation products of ENNB1 in HLM. This was also evident when comparing the sum of the areas under the concentration-time curve from 0 to 15 min. (AUC_0–15_) of M3/4, M5, and M6 to that of the parent compound, resulting in AUC_3/4 + 5 + 6_/AUC_ENNB1_ = 0.9, which confirmed them as major metabolites. Consequently, they were further used as the endpoints for experiments exploring ENNB1 biotransformation by phase I enzymes, particularly CYP3A, in more detail. All subsequent HLM assays were conducted with 15 min incubation time since M3/4 and M5 occurred shortly after incubation start and reached the highest signal intensities already after 5 min, whereas M6 showed the highest intensity after 10 min (Supplementary Figure S2). 

### 2.3. Role of CYP3A4/5 in ENNB1 Biotransformation

#### 2.3.1. Decrease of ENNB1 Metabolism with CYP3A4/5-Specific Inhibitor 

**Figure 2:**
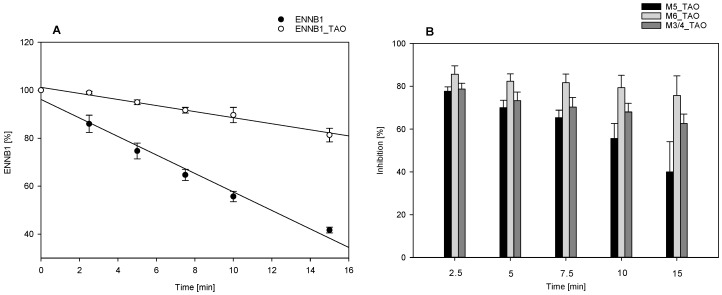
(**A**). Effect of CYP3A4/5 inhibition by troleandomycin (TAO) on ENNB1 depletion kinetics in human liver microsomes (HLM); (**B**). Inhibition of ENNB1 metabolite formation (M3/4, M5, M6) by TAO in HLM: the extent of inhibition was calculated at each time point from the ratio of peak areas for the individual metabolites with and without inhibitor ((1 − M_TAO_/M) × 100). All data points are expressed as mean ± SEM (*n* = 3) of three independent experiments.

The specific CYP3A4/5 inhibitor troleandomycin (TAO) was used to elucidate the importance of CYP3A4 for the biotransformation of ENNB1 in HLM. The assays were run under kinetic conditions with and without the inhibitor, and the impact of TAO on the formation of the major metabolites (M3/4, M5, M6) was investigated. In the absence of the inhibitor, about 42 ± 2% (mean ± SD, *n* = 3) of ENNB1 remained unmetabolised after 15 min. (Figure 2A). The ENNB1 metabolising efficiency slowed down when TAO was added to the assay, resulting in 81 ± 5% (mean ± SD, *n* = 3) of the compound remaining after 15 min of incubation in HLM. The elimination half-life (t_1/2_, _assay_) in the assay increased from 12 to 46 min (Figure 2A). 

The analysis of the metabolite profiles revealed that the formation of both the hydroxylated M3/4 and M5 and the carbonylated M6 was slowed down in the presence of the specific CYP3A4/5 inhibitor (Figure 2B), but with different effect sizes and time dependencies. While for M3/4 and M5, the TAO-mediated inhibition was maximal during the first 2.5 min of the incubation, reaching up to 84%, the effect lessened over time, declining to about 63% for M3/4 and 40% for M5 (Figure 2B). In contrast, the formation of M6 was inhibited with about 81% at all time points. Correspondingly, CYP3A4/5-inhibition by TAO reduced the AUC_0-15_ for M6 by a factor of 5.7 ± 2.5 (mean ± SD; *n* = 3), whereas the reduction was 3.3 ± 0.7 (mean ± SD; *n* = 3)-fold for M3/4 and 2.5 ± 0.6 (mean ± SD; *n* = 3)-fold for M5. Our results confirmed that ENNB1 is mainly oxidatively metabolised by CYP3A. It can thus be assumed that the inhibition of this biotransformation pathway also leads in vivo to a considerable increase of ENNB1 concentrations.

#### 2.3.2. ENNB1 as Inhibitor of Midazolam Biotransformation

MDZ is an important anaesthetic drug that is mainly metabolised in human liver by CYP3A4, 3A5, and 3A7, resulting in the production of the hydroxylated metabolites 1-hydroxy (1-OH) MDZ, 4-hydroxy (4-OH) MDZ, and 1,4-dihydroxy (1,4-OH) MDZ [25,26]. Therefore, MDZ is widely used as a model substrate for CYP3A in biotransformation pathway analysis [27]. When considering that ENNB1 is also metabolised by CYP3A, we investigated ENNB1′s potential for interactions with the MDZ metabolism in HLM in the present study. The inhibitory potency of ENNB1 was evaluated by comparing the half-life of MDZ depletion in the presence and absence of ENNB1, and regarded as an indicator for interferences with CYP3A-related reactions.

**Figure 3:**
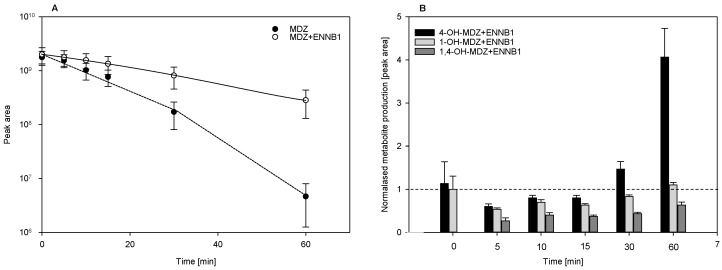
(**A**). Inhibition of midazolam (MDZ) depletion by ENNB1 in HLM. (**B**). Formation of hydroxylated MDZ metabolites in HLM in the presence of ENNB1 shown in comparison to incubations without ENNB1 (references set to 1). All data points are expressed as mean ± SEM (*n* = 3) of three independent experiments.

Under the assay conditions used (10 µM MDZ, 2 mg HLM), the MDZ depletion kinetics were nonlinear, showing that the overall MDZ clearance is a complex composition of several metabolic reactions (Figure 3A) [28]. The elimination half-life t_1/2, assay_ for MDZ in the linear phase of the assay (up to 15 min) was increased from 12 min to 27 min in the presence of 10 µM ENNB1 (Figure 3A). After 60 min incubation in HLM without or with the addition of ENNB1, respectively, about 0.2% or 12% of the initial MDZ were detectable. After 15 min of incubation, the fraction of unmetabolised MDZ increased by 23% in the presence of 10 µM ENNB1. Interestingly, the inhibitory effect of ENNB1 was already noticeable at a concentration of 5 µM, increasing the t_1/2, assay_ by 1.7 fold (data not shown). 

The analysis of the MDZ metabolites that were produced by HLM confirmed previous findings, showing that the formation of 1-OH-MDZ is preferred in comparison to 4-OH-MDZ and 1,4-OH-MDZ [27,28]. After 15 min of incubation, the signal intensity for 1-OH-MDZ was eight-fold (±3, *n* = 3) higher than that for 4-OH-MDZ (Supplementary Figure S3). The concentration-time curves of 1-OH-MDZ and 4-OH-MDZ formation linearly increased during the initial 15 min of incubation, whereas the formation of the double-hydroxylated 1,4-OH-MDZ was linear for up to 60 min (Supplementary Figure S3). When comparing the relative abundances (peak areas) of each metabolite in the presence and absence (reference value set to 1) of ENNB1 for each incubation time point revealed that metabolite formation was generally hindered during the first 15 min (Figure 3B). However, 4-OH-MDZ appeared to be considerably accumulated after 30 and 60 min, exceeding the level that was reached in ENNB1-free metabolism by a factor of 4. Addionally, the level of 1-OH-MDZ was returned to normal after 60 min, while the 1,4-OH-MDZ level was reduced to the half. Our results suggested that ENNB1 has the potential to inhibit CYP3A-catalysed biotransformation, as demonstrated for MDZ. 

The incubations of MDZ in the presence of DON in concentrations of up to 50 µM did not have a significant effect on the depletion kinetics (data not shown). Only an indication of a slight change in the metabolite formation profiles was observable.

### 2.4. DON Decreases the Formation of ENNB1 Metabolites in HLM and ND

**Figure 4:**
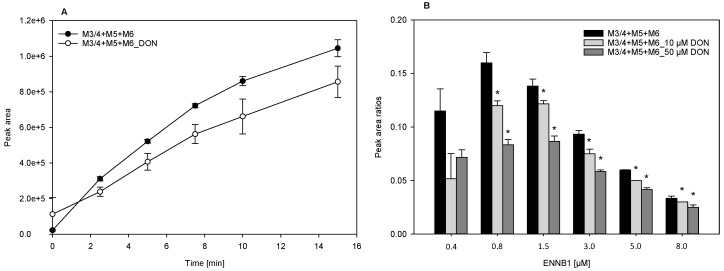
(**A**). DON (10 µM) decreases the formation of hydroxylated metabolites (M3/4 + M5 + M6) of ENNB1 (0.46 µM) in HLM. (**B**). Metabolite formation (summarised peak areas of M3/4, M5 and M6 in relation to peak area of ENNB1) at different start concentrations in ND assay after incubation for 15 min without and with DON (10 µM and 50 µM). *Statistically significant differences between experiments are marked (*P* < 0.05). Data points represent mean ± SEM (HLM: *n* = 3, ND: *n* = 6).

The potential interactions of ENNB1 with co-occurring DON were studied in HLM and in functional ND containing CYP3A4/5 and CYP3A4, respectively. DON is metabolised by conjugation with glucuronic acid (Phase II), so that direct competition at the acive binding site of the CYP enzyme is unlikely, but the indirect or allosteric inhibition might be possible, when considering the affinity of DON to proteins [18]. The optimal concentrations of ENNB1 and DON for the interaction study in HLM were determined in a pilot experiment with different ratios of DON and ENNB1 (DON/ENNB1 1:3; DON/ENNB1 1:1; and, DON/ENNB1 3:1) in 60 min incubations. A surplus of DON had the most noticeable impact on the metabolism of ENNB1 (data not shown), so that we increased the ratio and used 10 µM DON and 0.46 µM ENNB1 in all of the subsequent assays. DON inhibited the formation of the hydroxylated ENNB1 metabolites M3/4, M5, and M6 in HLM significantly, when the sum of the produced metabolites was considered (Figure 4A). In contrast, we did not observe a significant difference between the depletion of ENNB1 in the presence and absence of DON (data not shown).

The observed effect was further investigated in functional ND containing human CYP3A4 [29]. When different concentrations of ENNB1 were incubated for 15 min with DON, the formation of the hydroxylated metabolites was significantly lower than in assays only containing ENNB1 (Figure 4B). The effect was the strongest for the two lowest ENNB1 concentrations, but detectable at all levels. The relative metabolite formation (metabolite/ENNB1 peak areas) was the highest below 1.5 µM ENNB1 and it decreased steadily with increasing concentrations, both in the absence and presence of DON.

When considering these results, DON’s inhibitory potency on CYP3A4-dependent biotransformation was further examined with typical substrates.

#### 2.4.1. DON Decreases the Hydroxylation Efficiency of Progesterone and Atorvastatin Lactone in ND

The interaction of DON with CYP3A4-related biotransformation reactions was studied in more depth while using two important drugs, the steroid hormone progesterone (PGS) and the cholesterol-lowering statin atorvastatin (ATVS) lactone, which are mainly metabolised by this pathway [20,23]. The substances were incubated in functional ND with and without DON, and the formation of the respective CYP3A4-produced hydroxylation products 2β- and 6β-OH PGS or 2- and 4-OH ATVS lactone was measured. A comparison of the results indicated a small but reproducible inhibitory effect of increasing DON concentrations on the hydroxylation efficiency of both substrates (Table 2). The rate of PGS hydroxylation, while considering the sum of the produced metabolites in incubations with 15 µM and 40 µM, is reduced by about 15 to 20% in the presence of 49 µM DON. With regard to ATVS lactone hydroxylation, a decrease of the metabolite formation with about 20% was only observable for the minor product 2-OH ARVS lactone in incubations with 18 µM. These results confirm that DON shows a weak to moderate inhibition of the CYP3A4-dependent metabolism of the typical substrates. 

#### 2.4.2. Binding of DON and ENNB1 to CYP3A4 in Nanodiscs

**Figure 5:**
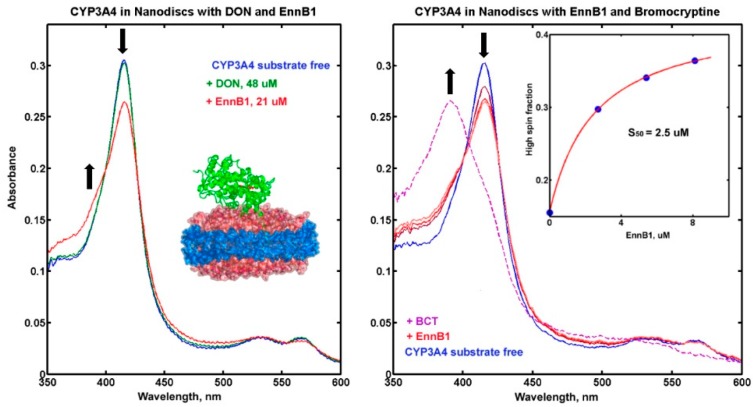
(**A**). UV-VIS spectra of CYP3A4-containing ND without substrate (blue) or after addition of DON (green) or ENNB1 (red). The spin shift from unbound low-state (down-arrow) to bound high-state (up-arrow) of the haem iron is indicated. (**B**). Spectra of functional ND without substrate (blue), with 2.7 µM, 5.4 µM and 8.1 µM ENNB1 (red), and after the addition of 5 µM bromocriptine (BCT, dashed magenta line). The spin shift of the haem iron from unbound low-state to bound high-state is marked with arrows. Insert - fitting of spin shift titration of ENNB1 with simple Langmuir isotherm and calculation of the midpoint at 2.5 µM, representing the ENNB1 concentration leading to 50% of the maximum effect (S_50_).

The functional ND system allows for the monitoring of substrate binding to CYP3A4 by measuring the transition of the haem iron from the unbound low-spin state at 420 nm to the bound high-spin state at 392 nm [30]. Different concentrations of ENNB1 and DON were used and the measured spectra were compared to that of substrate-free CYP3A4 (Figure 5A). Whereas a high DON concentration (48 µM) did not cause a noticeable spin shift, the addition of ENNB1 (21 µM) resulted in a visible spectral change corresponding to an increase of the high-spin state, which indicated binding. The ENNB1 concentration leading to 50% of the maximum effect (S_50_) was approximated to 2.5 µM in experiments with three different concentrations (2.7 µM, 5.4 µM, 8.1 µM) (Figure 5B). Moreover, the addition of the high-affinity CYP3A4 substrate BCT (5 µM) to the assay resulted in an almost complete spin shift to the high-spin state, which indicated that EnnB1 was reversibly bound to the enzyme and it could be displaced by BCT.

## 3. Discussion

ENNB1 is a biologically active cyclohexadepsipeptidic mycotoxin that is frequently detected in grain, sometimes at concentrations up to several mg/kg [1,2,3]. Although the risk for acute toxicosis from consumption of ENNB1-contaminated food products is considered as low [3], chronic dietary exposure to low levels of ENNB1 could have a negative impact on human and animal health due to its distinctive cytotoxic potential [5,6,7,8]. The co-occurrence of mycotoxins in food- and feedstuffs means dietary exposure to substance mixtures, which makes interactions at the reaction sites of target enzymes and receptors likely [11,13,14]. Furthermore, interrelations with drugs and endogenous metabolites can be expected. Besides the complexity of mixed effects, including the enhancement or reduction of toxicity, biotransformation pathways can also be compromised, when several substances compete for the same metabolic enzymes. In consequence, metabolite formation rates and essential kinetic parameters, such as the systemic elimination, can be changed. In the present study, we have therefore investigated the hepatic metabolism of ENNB1, estimating the kinetic characteristics and characterising the major metabolites. Moreover, we have estimated the potential for interaction with the important mycotoxin DON and several commonly used drugs by competitive binding, with the aim of elucidating the possible risks from exposure to substance mixtures. 

The kinetic parameters of ENNB1 were predicted by in vitro-to-in vivo extrapolation (IVIVE) based on substrate depletion assays in HLM. The calculated blood clearance for humans was comparable to that determined for the structurally closely related ENNB [24]. Moreover, the predicted maximum oral bioavailability (f_max_ = 45%) of ENNB1 was only slightly lower than that of ENNB. In contrast, animal studies showed that the bioavailabilities of both mycotoxins were with > 90% high in pigs [31,32] and with < 10% low in chicken [33]. Thus, the kinetic characteristics of ENNB1 and ENNB are apparently quite similar, but they differ strongly between species.

The main metabolites of ENNB1 biotransformation by HLM were identified as the products of hydroxylation, carbonylation, carboxylation, and oxidative methylation. They were identical with the ENNB1 metabolites that were determined in pig plasma after oral application [16] and homologous (+14 Da) to the previously described metabolites of ENNB from incubations in HLM [15]. The major metabolites were hydroxylated M3/4 and M5, as well as carbonylated M6, together accounting for 90% of the initial ENNB1 in a 15 min HLM assay. This high turnover indicated that ENNB1 is primarily metabolised by oxidative phase I enzymes, such as the P450 cytochromes, which were shown to be the main contributors of ENNB biotransformation [24]. In humans, CYP3A has a central role in the metabolism of xenobiotic drugs and contaminants [34]. With regard to the potential substrate interactions, it was therefore relevant to determine whether CYP3A was critically involved in ENNB1 metabolism and whether interactions with other substrates occurred.

While using the selective CYP3A4/5-inhibitor TAO in HLM incubations, the ENNB1 turnover decreased with about factor 4, which showed the importance of this pathway. When the inhibitory effect of ENNB1 on the CYP3A4-dependent metabolism of MDZ was reversely investigated, a significant decrease in the formation of MDZ metabolites was detectable. If this was caused by direct competition of both substrates in the reaction pocket of the enzyme or by allosteric changes in the CYP3A4-molecule from ligand binding to remote binding sites [35] could be determined in experiments with different ENNB1 concentrations. The biotransformation of MDZ in HLM resulted in the production of two major metabolites, as is well established [27]. The formation of 1-OH-MDZ was more affected by ENNB1 inhibition than that of 4-OH-MDZ, potentially because of the great difference in the reaction rates and enzyme affinities (K_M_,_1-OH-MDZ_ = 3.9 µM; K_M_,_4-OH-MDZ_ = 77.5 µM) [27]. Any ENNB1-caused decrease in the turnover capacity of the CYP3A4 would thus have a greater impact on the 1-hydroxylation, as observed in our study.

In contrast, the addition of DON (10 to 50 µM) did not change the metabolism of MDZ in HLM to the same extent (data not shown). DON is not subject to phase I metabolism, but is metabolised by phase II UDP-glucuronosyltransferases, so that a potential impact on CYP3A4 reactivity could only be induced by allosteric conformation changes. Our results are in agreement with the findings that were obtained in porcine hepatic microsomes, demonstrating that DON reduced CYP3A reactivity with about 20% [36]. Comparably, we showed that ENNB1 depletion in HLM was not noticeably slowed down by the presence of DON, but that a decrease of metabolite formation was detectable. However, the effect became significant when we studied DON and ENNB1 interaction in CYP3A4-containing functional ND, which indicated that DON might inhibit enzyme turnover by binding close to or at a certain distance of the reaction site [37].

Therefore, the potential of DON to interact with CYP3A4-dependent metabolic reactions was further analysed in ND with the typical substrates PGS and ATVS lactone that are both important drugs and used in hormone therapy and cholesterol reduction, respectively. We found that the formation of the major human CYP3A4 metabolites 2β-OH-PGS and 6β-OH-PGS [38] was decreased, showing that the unintentional dietary intake of DON could interfere with drug therapy. In contrast, the production of the minor metabolite 2-OH-ATVS lactone was reduced, but not that of the major metabolite 4-OH-ATVS lactone, which signifies a rather low impact of DON on the pharmacokinetics of this drug.

The binding affinity of ENNB1 to CYP3A4 was investigated in titration experiments in functional ND. The observed characteristic spin shift of the haem iron from unbound low-spin to bound high-spin state confirmed our results from the HLM experiments, which showed that ENNB1 is a substrate of CYP3A4. However, the affinity to the enzyme was considerably lower than that of BCT (Ks = 0.4 µM) [39], which was able to displace ENNB1 from the active site of the enzyme. As assumed from the inhibition experiments in HLM, DON did not cause any detectable spin shift, which confirmed that it did not bind to the reaction pocket of CYP3A4. The measurable effect of DON on CYP3A4-mediated metabolism of typical substrates and ENNB1 is thus probably caused by conformational changes from binding at the periphery of the enzyme. To our knowledge, the ability of DON to interfere with the CYP3A4-related metabolism was demonstrated for the first time in the present study. The analysis of the interactions between foodborne mycotoxins and medicinal drugs is still at the beginning, and our findings demonstrate that interference with kinetic processes should be considered.

## 4. Materials and Methods 

### 4.1. Materials

Enniatin B1 (ENNB1), deoxynivalenol (DON), midazolam (MDZ) and 4-hydroxymidazolam (4-OH-MDZ), mevastatine and bromocriptine (BCT), NADP^+^, NADPH, D-glucose 6-phosphate sodium salt, D-glucose 6-phosphate dehydrogenase from baker’s yeast (*Saccharomyces cerevisiae*), HEPES buffer, and ammonium formate (NH_4_COOH) were purchased from Sigma-Aldrich (Millipore Sigma; St. Louis, MO, USA). Troleandomycin (TAO) was supplied by Santa Cruz Biotechnology, Inc. (Dallas, Texas, TX, USA). Atorvastatin (ARVS) lactone and the metabolites 4-OH-ATVS lactone and 2-OH-ATVS lactone were from Toronto Research Chemicals (North York, ON, Canada), and cortexolone was from Steraloids (Newport, RI, USA). Progesterone (PGS) and the metabolite 6β-OH-PGS were from Clearsynth (Mississauga, Ontario, Canada). Fluka (Millipore Sigma; St. Louis, MO, USA) supplied magnesium chloride hexahydrate (MgCl_2_ × 6 H_2_O). With exception of DON that was dissolved in 90% acetonitrile (MeCN), all of the stock solutions were prepared in HPLC grade methanol (MeOH).

Fisher Scientific provided chromatographic solvents, including MeCN, MeOH, and water (Optima, LC/MS grade) (Waltham, MA, USA). Formic acid (HCOOH) was purchased from Merck (Darmstadt, Germany). 

### 4.2. Microsomal Incubations

#### 4.2.1. Performance of Metabolism Experiments

The in vitro experiments were performed with human liver microsomes (HLM Lot# IHG, SBM, YAO; Celsis, Baltimore, MD, USA), as previously described [16]. The microsomal assay mixture (final volume 1 mL) contained NADPH-generating system (0.91 mM NADPH, 0.83 mM NADP^+^, 19.4 mM glucose-6-phosphate, 1 U/mL glucose-6-phosphate dehydrogenase, and 9 mM MgCl_2_ × 6 H_2_O), incubation buffer (45 mM HEPES pH 7.4), and 2 mg microsomal protein. The biotransformation reactions were initiated by the addition of ENNB1 in MeOH. 

The microsomal incubations were conducted for the determination of four different endpoints: I) incubation conditions with regard to incubation time (up to 60 min) and substrate concentrations (ENNB1: 0.46 µM, 0.8 µM, 3 µM and 6 µM) were optimised, elimination constants were determined and used for the in vitro-to-in vivo extrapolation of kinetic parameters; II) the importance of cytochrome P450 (CYP) 3A4 in the biotransformation of ENNB1 was evaluated by using TAO (50 µM), which is a specific CYP3A4 inhibitor. The HLM were pre-incubated with TAO for 2 min. at 37 °C before ENNB1 (0.46 µM) was added. The differences in the half-life of ENNB1 depletion in the presence and absence of TAO were used to determine the inhibition potential, which was considered to be indicative for the extent of ENNB1-metabolism by CYP3A4; III) the inhibitory potential of ENNB1 on MDZ metabolism was investigated. A fixed concentration of MDZ (10 µM) was incubated in HLM in the presence of either ENNB1 (1 µM, 5 µM, and 10 µM) or DON (10 µM and 50 µM). The inhibitory potential of ENNB1 and DON on MDZ metabolism was assessed by determining the effects on MDZ depletion efficiency and on the formation of MDZ-related metabolites in the presence and absence of both mycotoxins; and, IV) the potential interaction/competition of ENNB1 and DON in HLM was studied by incubating ENNB1 (0.46 µM) in the presence and absence of DON (10 µM) for 15 min. to determine the effect on ENNB1 depletion.

All of the incubations were stopped with the addition of 130 µL ice-cold MeCN. The samples were kept on ice until they were centrifuged (Eppendorf AG, Hamburg, Germany) for 10 min at 20,000× *g* to precipitate proteins. The supernatants were collected, transferred to HPLC sample vials, and stored at −20 °C until analysis by liquid chromatography-iontrap mass spectrometry (LC-ITMS) or liquid chromatography-high-resolution mass spectrometry (LC-HRMS). Control incubations in HLM with no cofactor, no microsomal protein, and/or no substrate were performed to validate CYP P450 enzyme-dependent metabolism. All of the experiments were performed in duplicate on at least three different days.

#### 4.2.2. In Vitro-to-In Vivo Extrapolation of Kinetic Parameters

The kinetic parameters were derived in HLM assays from the substrate depletion rate constants (k_e_) that were determined by regression analysis of measured peak areas of the added substrate versus time curves (A(t) = b + a*e^−ket^), as described before [24]. Briefly, the assay half-lifes (t_1/2, assay_ = ln2/k_e_) and assay clearances (CL_assay_ = V_assay_*k_e_) were calculated under consideration of the assay volume (V_assay_). Disregarding the potential protein binding of the substrate in the reaction mixture (assuming that the fraction unbound in the assay (f_u, assay_ ~ 1)), the determined assay clearances approximated the intrinsic assay clearances (CL_int, assay_), which can be described by the Michaelis–Menten equation parameters maximal velocity (v_max, assay_) and reaction constant (K_M, assay_) under the condition that the substrate concentration is well below the K_M_-value (CL_int, assay_ = v_max, assay_/K_M, assay_). The K_M, assay_ for ENNB1 depletion was determined with different initial concentrations by plotting the determined depletion rate constants versus the respective concentrations. The inflection point of the curve in a lin-log plot represented the K_M_-value, which occurred when k_e_ is half of the theoretical maximum k_0_ at infinitesimally low ENNB1 concentrations (k_e_ = k_0[ENNB1]→0_ *(1 − [ENNB1]/([ENNB1] + K_M_)) [40].

The CL_int, assay_ in HLM was upscaled to the assay-independent, intrinsic liver clearance (CL_int_ = CL_int, assay_*MRI*RLW/Prot_assay_) by considering the amounts of microsomal protein in the assay (Prot_assay_), human relative liver weight (RLW), and human microsomal recovery index (MRI) (Table 1). In vitro-to-in vivo extrapolation (IVIVE) was performed by applying the well-stirred liver model [41] and calculating the systemic blood clearances (CL_b_) from the CL_int_, while considering the human hepatic blood flow (Q) (CL_b_ = Q*CL_int_*f_u,b_/(Q + CL_int_*f_u,b_)). The fraction unbound (f_u,b_) in blood of ENNB1 was not considered for the calculation of CL_b,vitro_ (f_u,b_ ≈ 1) in this approximation, although f_u,b_ is possibly substantially lower than 1 for this lipophilic compound. The maximal bioavailability (f_max_) after oral application was calculated under the assumption of complete absorption from the gastrointestinal tract (f_a_ = 1) as f_max_ = 1 − CL_b_/Q.

### 4.3. Incubations in Nanodiscs

#### 4.3.1. Protein Expression and Purification of Functional Nanodiscs

Expression in *E. Coli*, purification of recombinant membrane scaffold protein (MSP1D1), human hepatic CYP3A4 and rat NADPH-dependent CYP P450-reductase (CPR), and preparation of CYP3A4-containing 1-palmitoyl-2-oleoyl-*sn*-glycero-3-phosphocholine (POPC) nanodiscs (ND) were performed, as described previously [21,22,42,43]. CYP3A4 was available as a NF-14 gene construct in the pCWOri^+^ vector with a C-terminal penta-histidine affinity tag that was generously provided by Dr. F. P. Guengerich (Vanderbilt University, Nashville, TN, USA). Full length CPR was expressed while using a rat CPR/pOR262 plasmid, which was a generous gift from Dr. Todd D. Porter (University of Kentucky, Lexington, KY, USA). CYP3A4 was incorporated in ND from assembly mixture containing CYP3A4, MSP1D1 and POPC solubilized in cholate, as described [22,44,45]. The detergents were removed by incubation with Amberlite XAD-2 (Millipore Sigma, Saint-Louis, MO, USA) for at least 4 h on ice. Further purification steps included nickel-affinity chromatography (Ni-NTA, Thermo Scientific, Schaumburg, IL, USA), followed by size-exclusion chromatography (Superdex 200HR10/30; GE Life Sciences, Chicago, IL, USA), as described before [22]. CPR was incorporated by direct addition to CYP3A4 ND at 4:1 molar access for functional studies, as described [46].

#### 4.3.2. Titration of Substrate Binding to CYP3A4/CPR-ND

Substrate binding was studied in titration experiments with 600 µL of 3 µM CYP3A4 in functional ND. Binding was monitored by measuring absorption spectra of the CYP3A4 in ND in the range of 330 to 750 nm while using a Cary Bio 300 spectrophotometer (Varian, Palo Alto, CA, USA) at 37 °C. Stock solutions of 3.0 mM ENNB1 and 3.4 mM DON in MeOH were used in titration experiments with the final MeOH concentration in assay not exceeding 2%. Substrate binding was monitored by changes in the absorption spectra that were caused by the transition from the low-spin (417 nm; unbound) to the high-spin (392 nm; substrate-bound) state of the CYP3A4 haem iron. The measured spectra were corrected for dilution factors and processed with a singular value deconvolution subroutine that was written in MATLAB (Math-Works, Natick, MA, USA) for the number of linear independent spectral components, as described [21]. The second singular vector was used for fitting, while using the Langmuir equation. The calculated midpoint of the isotherm was used as an estimate for the substrate concentration, leading to 50% of the maximum effect (S_50_). The high-affinity CYP3A4-substrate BCT (5 µM) was added to ENNB1-saturated ND at the end of the titration and spectra of CYP3A4-ND in the range of 330 to 750 nm were measured in order to confirm the reversible binding of ENNB1 to fully functional CYP3A4.

#### 4.3.3. Interference of DON with the Metabolism of Typical CYP3A4-s Substrates in Functional ND

The impact of DON on PGS hydroxylation and ARVS lactone hydroxylation was examined in CYP3A4-containing ND.

Metabolism experiments with 15 and 40 µM PGS were carried out with functional ND in 1 mL 100 mM HEPES buffer (pH 7.4; 10 mM MgCl_2_; 0.1 mM dithiothreitol) in a spectrophotometer cuvette. The reaction was started by the addition of 200 nmol NADPH, and NADPH consumption was monitored at 340 nm (extinction coefficient 6.22 mM^−1^cm^−1^) [23]. After 5 min, 0.5 mL aliquots were removed from the cuvette, mixed with 2 mL dichloromethane, and then used for the analysis of the metabolites 2β-OH-PGS and 6β-OH-PGS, as described [20]. Briefly, cortexolone (2.5 nmol per sample) was added as the internal standard (IS) and the samples were thoroughly mixed. After phase separation, the organic layer was isolated and the solvent was removed under a stream of nitrogen. The samples were re-dissolved in 70 μL MeOH, and 40 μL were analysed by high-resolution liquid chromatography (HPLC) on an Ace 3 C18 column (2.1 mm × 150 mm) (MAC-MOD Analytical, Chadds Ford, PA, USA) while using an LC-20AD HPLC system with UV detector (Shimazu Scientific, Columbia, MD, USA). The mobile phase consisted of water (A) and MeCN/MeOH (50:50) (B). Products of PGS hydroxylation were separated by a linear gradient that started at 15% MeCN/ MeOH and rising to 37% within 35 min at a flow rate of 0.2 mL/min. Absorbance was measured at 240 nm and the signals were calibrated while using the commercially available PGS metabolite 6β-OH-PGS. Data were processed while using Millennium^TM^ software (Waters Corporation, Milford, MA, USA).

Metabolism experiments with 8 and 18 µM ARVS lactone were comparably performed. After incubation for 5 min, 0.2 mL aliquots were quenched with 1.6 mL MeCN/MeOH (2:1) supplemented with the IS mevastatine, and dried. The samples were dissolved in 100 µL MeOH and 30 µL were analysed by HPLC, as described above. UV-absorbance was measured at 240 nm and signals were calibrated using the commercially available ARVS lactone metabolites 4-OH-ATVS lactone and 2-OH-ATVS lactone, and the data were processed using Millennium^TM^ software.

All of the experiments were performed at least in duplicate and in the absence or presence of DON (10 µM, 49 µM). DON was added to the cuvette together with PGS or ARVS lactone. The mixture was briefly stirred before NADPH was added to start the reaction. Three time points were taken at 4, 5, and 6 min, and the results were averaged.

### 4.4. Liquid Chromatography-Iontrap Mass Spectrometry (LC-ITMS)

ENNB1 and its major biotransformation products were analysed while using a Finnigan LTQ linear ion trap mass spectrometer (Thermo Fisher Scientific Inc., Waltham, MA, USA) that was coupled to an ACQUITY UPLC System (Waters). Separation was achieved on a 50 × 2.1-mm i.d. C18 3-μm SunFire column (Waters) with 0.5 μm pre-column filter (Supelco, Bellefonte, PA, USA). The mobile phase was composed of water (A) and MeCN (B) (both containing 2 mM NH_4_COOH and 2 mM HCOOH). The optimised linear gradient started at 40% B and was held for 1.5 min, before it was raised to 70% A over 14.5 min. The column was flushed with 100% A for 2 min, returned to 40% B, and equilibrated for 2.0 min. The total run time was 20 min.

The samples were analysed in the positive ionisation mode. The parameters of the ESI interface were adjusted, as follows: spray voltage 4.5 kV, capillary temperature 275 °C, tube lens offset 105 V, sheath gas rate 39 L/min, and auxiliary gas rate 10 L/min. Multiple-stage MS^n^ fragmentation (collision-induced dissociation (CID)) was performed while using an isolation width of 2 *m/z* for precursor ions. The activation energy (Q) value was set to 0.25, the activation time to 30 ms, and the collision energy to 35 eV.

ENNB1 and its putative metabolites were identified by their molecular weights (mass-to-charge (*m/z*) ratios) and specific retention times. In addition, the MS^n^ data of the sodiated molecular ions were specified in fragmentation experiments and used for confirmation [16]. The measured peak areas of the ammoniated molecular ions ([M+NH_4_]^+^) were used for the determination of in vitro kinetic parameters and for quantification purposes.

### 4.5. Liquid Chromatography-High Resolution Mass Spectrometry (LC-HRMS)

The inhibition of MDZ biotransformation with ENNB1 was analysed by LC-HRMS while using a QExactive^TM^ Hybrid Quadrupole-Orbitrap mass spectrometer that was equipped with a heated electrospray ion source (HESI-II) and coupled to a Vanquish UHPLC system (Thermo Fisher Scientific). The HESI-II interface was operated at 300 °C in negative and positive ionisation mode while using fast polarity switching in the mass range *m/z* 100–500, and the parameters were adjusted, as follows: spray voltage 3.2 and 2.8 kV (positive and negative mode, respectively), capillary temperature 280 °C, sheath gas flow rate 35 L/min., auxiliary gas flow rate 10 L/min., and S-lens RF level 55. Data were acquired in the full scan monitoring (FS)/data-dependent MS^2^ (dd-MS^2^) mode targeting the [M+H]^+^ ions for MDZ (*m/z* 326.0855), 1-OH-MDZ and 4-OH-MDZ (*m/z* 342.0834), and 1,4-OH-MDZ (*m/z* 358.0753). The FS data were acquired at a mass resolution of 70,000 full width half-maximum (FWHM) at *m/z* 200, while mass resolution was set to 17,500 FWHM at *m/z* 200 during dd-MS^2^. The automated gain control (AGC) target was set to 1 × 10^6^ ions for a maximum injection time (IT) of 250 ms in the FS mode, whereas the AGC target was 1 × 10^5^ for dd-MS^2^ mode and the IT was 100 ms.

Chromatographic separation was performed at 30 °C on a 150 × 2.1 mm i.d. Kinetex F5 LC column (2.6 μm; Phenomenex) with 0.5 μm × 0.004 in. ID HPLC KrudKatcher Ultra Column In-Line filter (Phenomenex). The mobile phase consisted of water (A) and MeCN (B) containing 0.1% formic acid. Separation was achieved while using a gradient that ranged from 20% B at 1 min. to 80% B at 20 min. and further to 95% B at 21 min. at a flow rate of 0.25 mL/min. After flushing the column for 2 min. with 95% B, the mobile phase composition was returned to the initial conditions, and the column was isocratically eluted for 3.0 min. The total run time was 26.5 min.

Xcalibur software was used for instrument control and calculation of mass errors and elemental compositions. The metabolites were identified based on compound-specific retention times, comparison to a reference standard (4-OH-MDZ), fragmentation patterns, and accurate masses, which were obtained while using a mass window of ± 5 ppm with respect to the theoretical accurate masses. Measured peak areas were used for semi-quantification of the results.

### 4.6. Data Analysis and Statistics

Statistical comparisons of mean values of multiple analyses were performed while using one-way analysis of variance (ANOVA) or Student *t*-test (Sigma Plot version 13.0; JMP version 5.0a, SAS Institute, Inc., Cary NC, USA). *P* < 0.05 was considered as statistically significant.

## 5. Conclusions

The mycotoxin ENNB1 was studied in HLM and CYP3A4-containing functional ND with the aim of elucidating kinetic characteristics, the main biotransformation pathway and major metabolites, and the interaction potential with other substrates. ENNB1 was predicted to be eliminated with half the efficiency of the human liver blood flow. Eleven metabolites were identified, which included products of hydroxylation, carbonylation, carboxylation, and oxidative methylation reactions. ENNB1 was mainly metabolised by CYP3A4/5, and specific inhibitors could inhibit the turnover. Although the mycotoxin DON did not bind to the active site of CYP3A4, it slowed ENNB1 metabolite formation, which indicated that it could affect the metabolism through conformational changes of the enzyme. At the same time, DON had the potential to decrease the metabolism efficiency for other CYP3A4/5 substrates, such as the important drugs progesterone and atorvastatin lactone. The results emphasise the importance of drug−drug interaction studies, also with regard to natural toxins.

## Figures and Tables

**Table 1:** Kinetic parameters of ENNB1 in humans by in vitro-to-in vivo extrapolation from assays with human liver microsomes.

Parameter	HLM *
k_e_ [min^−1^] ^a^	0.065
t_1/2, assay_ [min]	11
K_M, assay_ [µM] ^b^	10.1
CL_int, assay_ [mL/min]	0.07
CL_int_ [L/(h×kg×BW)]	1.72
CL_b_ [L/(h×kg×BW)]	0.77
f_max_ (%)	45

^a^ ENNB1 concentration 3 µM; ^b^ derived from depletion assays with three ENNB1 concentrations (0.8; 3; 6 µM); * microsomal recovery index (MRI) = 40 mg/g, relative liver weight (RLW) = 22 g/kg bodyweight (BW), liver blood flow (Q _human_) = 1.4 L/(h × kg).

**Table 2:** Decrease of progesterone (PGS) and atorvastatin (ATVS) lactone hydroxylation by DON in CYP3A4-containing nanodiscs (ND).

Substrate (µM)	Without DON ^1^	DON ^1^, 10 μM	DON ^1^, 49 μM
	nmol/(min×nmol CYP3A4)	nmol/(min×nmol CYP3A4)	nmol/(min×nmol CYP3A4)
PGS^#^, 15 μM	5.3 ± 0.6	5.0 ± 0.6	4.1 ± 0.4
PGS^#^, 40 μM	10.4 ± 1.0	9.8 ± 0.9	8.9 ± 0.8
ATVS lactone*, 8 μM	2.8 ± 0.2/0.47 ± 0.08	2.7 ± 0.25/0.48 ± 0.08	2.7 ± 0.16/0.52 ± 0.06
ATVS lactone*, 18 μM	3.2 ± 0.2/1.1 ± 0.1	3.2 ± 0.2/0.97 ± 0.12	3.1 ± 0.18/0.87 ± 0.13

^1^ Data points are expressed as mean ± SEM (*n* = 3, with two technical replicates). #Formation rates were calculated for the sum of the hydroxylated metabolites 2β- and 6β-OH-PGS. * Formation rates were separately calculated for the hydroxylated metabolites 4-OH-ATVS lactone (major product) and 2-OH-ATVS lactone (minor product).

## Supplementary Materials

The following are available online at https://www.mdpi.com/2218-1989/9/8/158/s1, Figure S1: Representative LC-ITMS chromatograms of extracted ammoniated molecular ions at *m/z* 687 (A), *m/z* 685 (B), and *m/z* 701 (C) of the putative ENNB1 metabolites M1 to M11 detected after 15 min. incubation with HLM; Figure S2: Formation of the putative ENNB1 metabolites M2 to M6 and M8 to M11 in HLM. Results are expressed as mean peak areas of three independent microsomal incubations. M7 was not included because of low signal intensity; Figure S3: Formation of the major MDZ metabolites 1-OH-MDZ, 4-OH-MDZ and 1,4-OH-MDZ. Results are expressed as mean peak areas of three independent microsomal incubations; Table S1: Molecular ions of EnnB1 and EnnB1-related metabolites by LC-ITMS.
