# Supplementary material for: Biotransformation of the Mycotoxin Enniatin B1 by CYP P450 3A4 and Potential for Drug-Drug Interactions
Source: Metabolites. 2019 Jul 27;9(8):158. doi: 10.3390/metabo9080158 (PMC6724072; doi:10.3390/metabo9080158)
Supplement: Supplementary file 1 [file metabolites-09-00158-s001.pdf]

Supplementary Material

Biotransformation of the Mycotoxin Enniatin B1 by CYP P450 3A4 and Potential for Drug-Drug Interactions

Lada Ivanova [1,*], Ilia G Denisov [2], Yelena V Grinkova [2], Stephen G Sligar [2] and Christiane K Fæste [1]

[1] Chemistry and Toxinology Research Group, Norwegian Veterinary Institute, P.O. Box 750 Sentrum, 0106 Oslo, Norway
[2] Department of Biochemistry, University of Illinois, Urbana, IL 61802, USA
* Correspondence: lada.ivanova@vetinst.no; Tel.: +47-9151842

Table of contents

**Table S1.** Molecular ions of EnnB1 and EnnB1-related metabolites by LC-ITMS.	S3
**Figure S1.** Representative LC−ITMS chromatograms of extracted ammoniated molecular ions at *m/z* 687 (A), *m/z* 685 (B), and *m/z* 701 (C) of the putative ENNB1 metabolites M1 toM11 detected after 15 min incubation with HLM .	S4
**Figure S2.** Formation of the putative ENNB1 metabolites M2 toM6 and M8 toM11 in HLM. Results are expressed as mean peak areas of three independent microsomal incubations. M7 was not included because of low signal intensity.	S5
**Figure S3.** Formation of the major MDZ metabolites 1-OH-MDZ, 4-OH-MDZ and 1,4-OH-MDZ. Results are expressed as mean peak areas of three independent microsomal incubations.	S6

Table S1. Molecular ions of ENNB1 and eleven putative hepatic metabolites in LC-ITMS.

Metabolite	*Molecular formula*	*Observed mass [M+Na]⁺*	*Observed mass [M+NH4]⁺*	*Biotransformation pathway*
EnnB1	C34H59N3O9	676.4	671.4	
M1	C34H59N3O10	692.4	687.4	Oxydative demethylation
M2−M5	C34H59N3O10	692.4	687.4	Hydroxylation
M6−M8	C34H57N3O10	690.4	685.4	Carbonylation
M9−M11	C34H57N3O11	706.4	701.4	Carboxylation

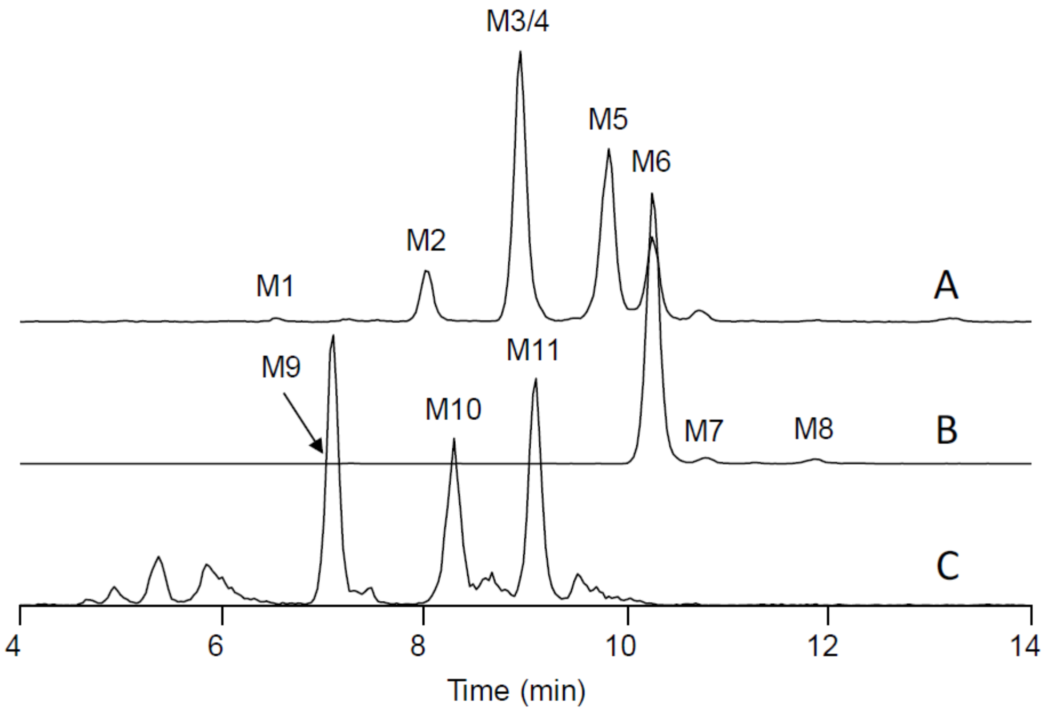

Figure S1. Representative LC−ITMS chromatograms of extracted ammoniated molecular ions at *m*/*z* 687 (A), *m*/z 685 (B), and *m*/*z* 701 (C) of the putative ENNB1 metabolites M1 to M11 detected after 15 min incubation with HLM.

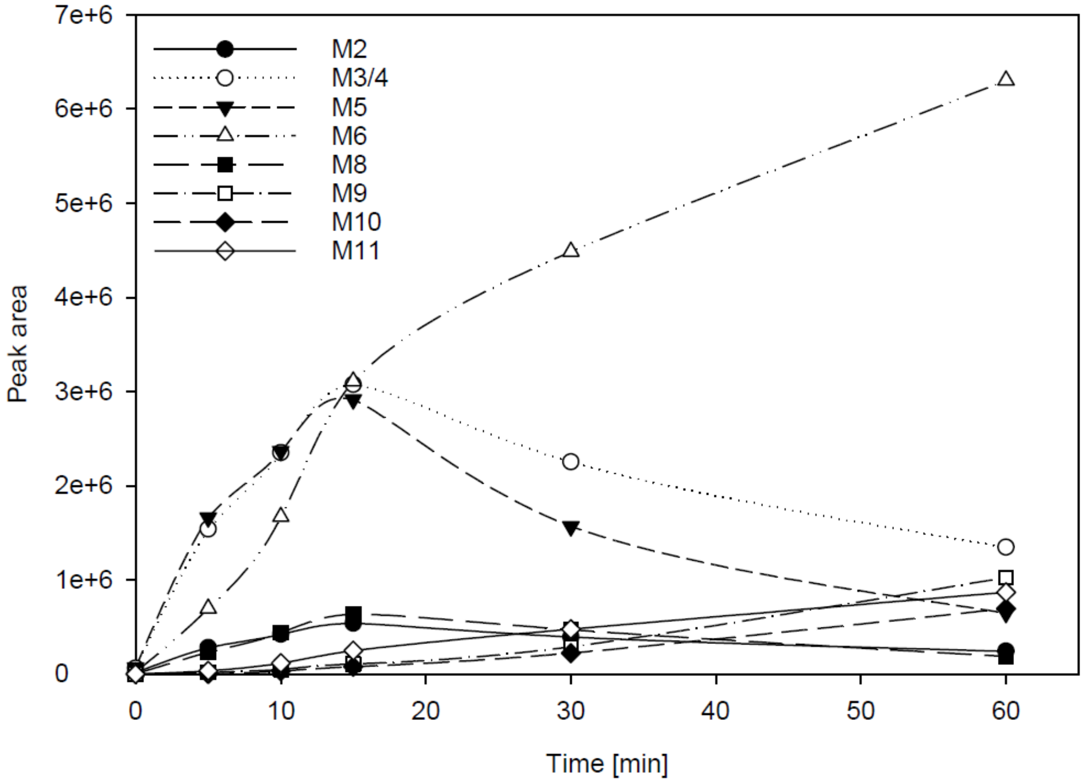

Figure S2. Formation of the putative ENNB1 metabolites M2 toM6 and M8 to M11 in HLM. Results are expressed as mean peak areas of three independent microsomal incubations. M7 was not included because of low signal intensity.

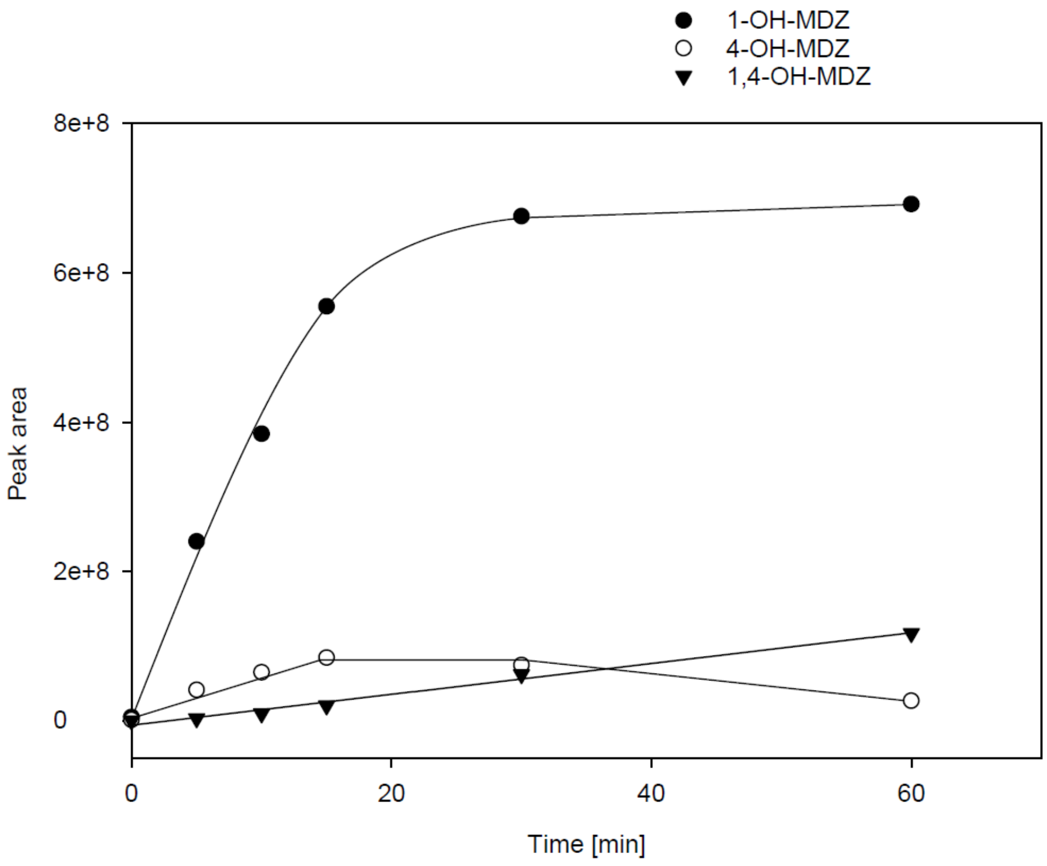

Figure S3. Formation of the major MDZ metabolites 1-OH-MDZ, 4-OH-MDZ and 1,4-OH-MDZ. Results are expressed as mean peak areas of three independent microsomal incubations.
